# Symptom Improvement in a Patient With Chronic Pronator Teres Syndrome Treated With Ultrasound-Guided Hydrodissection and Manual Therapy: A Case Report

**DOI:** 10.7759/cureus.95978

**Published:** 2025-11-03

**Authors:** Yusuke Nishimaki, Masashi Kawabata, Atsuro Yamazaki, Wataru Iwamoto

**Affiliations:** 1 Department of Rehabilitation, Kurage Orthopedic Clinic, Chiba, JPN; 2 Department of Rehabilitation, Kitasato University School of Allied Health Sciences, Sagamihara, JPN; 3 Department of Orthopedics, Kurage Orthopedic Clinic, Chiba, JPN; 4 Department of Sports Medicine, Edogawa Hospital, Tokyo, JPN

**Keywords:** manual mobilization techniques, median nerve entrapment, peripheral nerve restrictions, rehabilitation exercises, upper extremity neuropathy

## Abstract

Pronator teres syndrome (PTS) is an uncommon neuropathy caused by compression of the median nerve at the proximal forearm, resulting in paresthesia and pain in the radial digits. Although conservative management is typically the preferred initial approach, chronic and refractory cases often require multimodal treatment strategies.

A 55-year-old female truck driver presented with a five-year history of persistent bilateral numbness and pain radiating from the palms to the distal phalanges. Physical examination revealed positive pronator compression and bilateral upper limb neurodynamic tests, with a numerical rating scale (NRS) score of 8/10. Palpation during ultrasound imaging demonstrated localized tenderness and radiating pain around the pronator teres and median nerve. Cervical pathology and carpal tunnel syndrome were excluded.

The patient underwent ultrasound-guided hydrodissection of the median nerve at the pronator teres level every two weeks, combined with manual mobilization and a prescribed home-based exercise program. Symptomatic relief was achieved after the first session, and after four treatment sessions over two months, the NRS score improved from 8/10 to 3/10. Functional recovery was achieved, and the patient successfully maintained symptom control through continued adherence to the home-based exercise program.

This report highlights the potential effectiveness of a multimodal strategy for addressing chronic PTS. Ultrasound-guided hydrodissection may improve mobility of the median nerve by reducing perineural restrictions, while manual mobilization and gliding exercises are likely to provide complementary therapeutic effects. Given the chronicity of the patient’s condition, the early and sustained response underscores the potential utility of combining these techniques in refractory cases. Ultrasound-guided hydrodissection, when combined with manual therapy and a structured home-based exercise program, may represent a minimally invasive and effective management option for chronic pronator teres syndrome that is unresponsive to conservative care.

## Introduction

Pronator teres syndrome (PTS), which was first described by Seyffarth in 1951 [1], is a rare entrapment neuropathy of the median nerve in the proximal forearm. Although uncommon, PTS is more frequently observed in women in their 40s and 50s, and it is often associated with occupations requiring repetitive forearm and wrist movements [2]. Typical symptoms include proximal forearm pain and paresthesia radiating to the thumb, index finger, middle finger, and radial half of the ring finger [3]. Anatomical variations between the pronator teres muscle and the median nerve have been reported [4]. A cadaveric study of 100 forearms revealed that the humeral and ulnar heads of the pronator teres were present in 86 forearms. The median nerve passed between the two heads in 72, penetrated the ulnar head in 11, and coursed posteriorly to both heads in three. In forearms that did not have the ulnar head, the nerve passed posteriorly to the humeral head in 11 and penetrated it in three [4]. These variations may narrow the passageway and increase susceptibility to PTS. Differentiation from carpal tunnel syndrome (CTS) can be challenging due to overlapping symptoms; however, the absence of nocturnal pain can be a valuable diagnostic clue [5-9]. Electromyography and nerve conduction studies are frequently inconclusive [5-7]. Typical findings include tenderness over the pronator teres, reproduction of symptoms with repetitive pronation, and exacerbation during elbow flexion or resisted flexor digitorum superficialis contraction [5-7,10]. Conservative management, including rest, activity modification, physical therapy, and ultrasound-guided interventions, is considered the first-line approach, with reported success rates ranging from 29% to 70% [11]. Surgical decompression, including wide open release, limited open release, or minimally invasive endoscopic techniques, is reserved for refractory cases [3,12]. Recent reports have also emphasized the potential role of ultrasound-guided hydrodissection as an emerging minimally invasive option for managing peripheral nerve entrapments, including pronator teres syndrome [11,13]. Here, we describe a case of chronic PTS that showed early and substantial improvement following a multimodal treatment approach combining ultrasound-guided hydrodissection, manual mobilization, and nerve gliding exercises. The objective of this report is to present the therapeutic process and clinical outcome of this patient, and to highlight the potential role of ultrasound-guided, minimally invasive interventions as part of comprehensive rehabilitation for chronic PTS.

## Case presentation

A 55-year-old female truck driver presented with a five-year history of persistent bilateral numbness and pain radiating from the palms to the distal phalanges. The paresthesia and pain were distributed along the thumb, index, and middle fingers, corresponding to the median nerve dermatome. The symptoms were aggravated by repetitive forearm pronation and cargo handling, and relieved by rest.

On examination, the pronator compression test and upper limb tension test 1 were bilaterally positive, with an NRS score of 8/10. Jackson compression test and Spurling test were negative, excluding cervical radiculopathy. Similarly, Phalen’s test and carpal compression tests were negative, ruling out CTS. Manual muscle testing revealed no deficits in the flexor digitorum superficialis, flexor pollicis longus, or pronator quadratus. Grip strength, measured using a Smedley-type hand dynamometer (ES-100, model EKJ080; Evernew Inc., Tokyo, Japan), was 28 kg (right, R) and 25 kg (left, L).

Ultrasound-guided palpation demonstrated localized tenderness and reproduction of radiating pain around the pronator teres and median nerve at that level. Palpation away from the nerve failed to reproduce the symptoms, strongly suggesting entrapment at the pronator teres level (Table 1, Videos 1-2). As this is a single-patient case report, no formal blinding was applied. To minimize assessment bias, pain intensity and physical tests were conducted using standardized procedures and consistent numerical rating scale (NRS) scoring. Ultrasound assessments were performed by the same experienced clinician using consistent probe placement and imaging parameters across sessions to ensure procedural reproducibility as much as possible.

**Table 1:** Baseline clinical findings and outcomes in the presented case Tests that were negative at baseline were not repeated at follow-up. Patient-reported outcome measures were not collected. NRS, numerical rating scale; ULNT, upper limb neurodynamic test; MMT, manual muscle testing; FDS, flexor digitorum superficialis; FPL, flexor pollicis longus; PQ, pronator quadratus; R, right; L, left

Category	Test	Initial findings	Post-treatment findings
Patient characteristics	Age	55 years	ー
	Sex	Female	ー
	Occupation	Truck driver	ー
	Duration of symptoms	5 years	ー
Symptom intensity	NRS (0/10)	8/10	3/10
Diagnostic tests	Pronator compression test (NRS: /10)	Positive, 8	Negative, 0
	Pain during ULNT (NRS: /10)	Positive, 8	Negative, 0
	Jackson compression test	Negative	Not reassessed
	Spurling test	Negative	Not reassessed
	Phalen’s test	Negative	Not reassessed
	Carpal compression test	Negative	Not reassessed
MMT (Grade 0 to 5)	FDS	5	Not reassessed
	FPL	5	Not reassessed
	PQ	5	Not reassessed
Grip strength	Dynamometer	28 kg (R), 25 kg (L)	Not reassessed
Ultrasound-guided palpation	Local tenderness and radiating pain at pronator teres level (NRS: /10)	8/10	0

**Video 1:** Ultrasound-guided palpation of the Median Nerve at the Pronator Teres Level Real-time ultrasound-guided palpation of the median nerve at the pronator teres level. Localized tenderness and reproduction of radiating pain were observed, findings suggestive of entrapment at this site. Short explanatory text captions have been added within the video to indicate the maneuver and the observed clinical finding.

**Video 2:** Model Demonstration of Palpation of the Median Nerve at the Pronator Teres Level Model demonstration of palpation of the median nerve at the pronator teres level. This video, obtained from a healthy volunteer, illustrates the ultrasound-guided palpation technique for educational purposes and does not represent the actual patient described in this report.

Intervention

The patient presented with bilateral symptoms, and ultrasound-guided hydrodissection combined with manual mobilization was performed bilaterally at the pronator teres level following the same standardized protocol. The procedures were conducted sequentially during each session. The patient underwent treatment sessions once every two weeks, each consisting of ultrasound-guided hydrodissection of the median nerve at the pronator teres level, followed by targeted manual mobilization of the interfascial plane between the pronator teres and the median nerve.

Hydrodissection of the median nerve was performed under ultrasound guidance at the level of the pronator teres. The procedure was performed by a licensed physician using a 27-gauge needle under real-time ultrasound guidance with an out-of-plane approach. A solution consisting of 1 mL of triamcinolone acetonide (Kenacort® 40 mg/mL), 1% mepivacaine, and 20 mL of normal saline was prepared. Approximately 5 mL of this mixture was injected per session at the pronator teres level to separate the perineural fascial interface, with continuous visualization of the needle tip to ensure procedural safety. A small volume of normal saline was injected in-plane to release perineural fascial restrictions and enhance nerve mobility [12]. This technique aimed to restore the natural gliding dynamics of the median nerve within the anatomical tunnel.

Following hydrodissection, meticulous manual mobilization of the pronator teres and adjacent soft tissues was performed under continuous real-time ultrasound visualization to further enhance neural decompression and optimize interfascial mobility (Videos 3-4). Manual mobilization was performed under real-time ultrasound visualization with gentle-to-moderate fingertip pressure, adjusted according to the patient’s comfort level and tenderness response.

**Video 3:** Ultrasound-Guided Manual Mobilization of the Median Nerve at the Pronator Teres Level This video demonstrates ultrasound-guided manual mobilization of the median nerve at the pronator teres level. The maneuver involves inserting the examiner’s finger alongside the pronator teres muscle, as indicated by the arrow, and applying gentle pressure in the direction shown. This technique is intended to enhance median nerve gliding and reduce perineural restrictions. This demonstration shows the role of real-time ultrasound guidance in performing safe and targeted manual mobilization.

**Video 4:** Model Demonstration of Manual Mobilization Between the Pronator Teres and the Median Nerve Model demonstration of manual mobilization between the pronator teres and the median nerve. This video, obtained from a healthy volunteer, illustrates the ultrasound-guided mobilization technique for educational purposes.

The number of gliding movements was not strictly predetermined and was pragmatically adjusted based on the degree of tissue tension and patient tolerance, typically involving several short-axis movements per side. Each manual mobilization session lasted approximately 3-5 minutes in total. The procedure followed a consistent concept of ultrasound-guided interfascial mobilization for median nerve entrapment [12].

**Figure 1:**
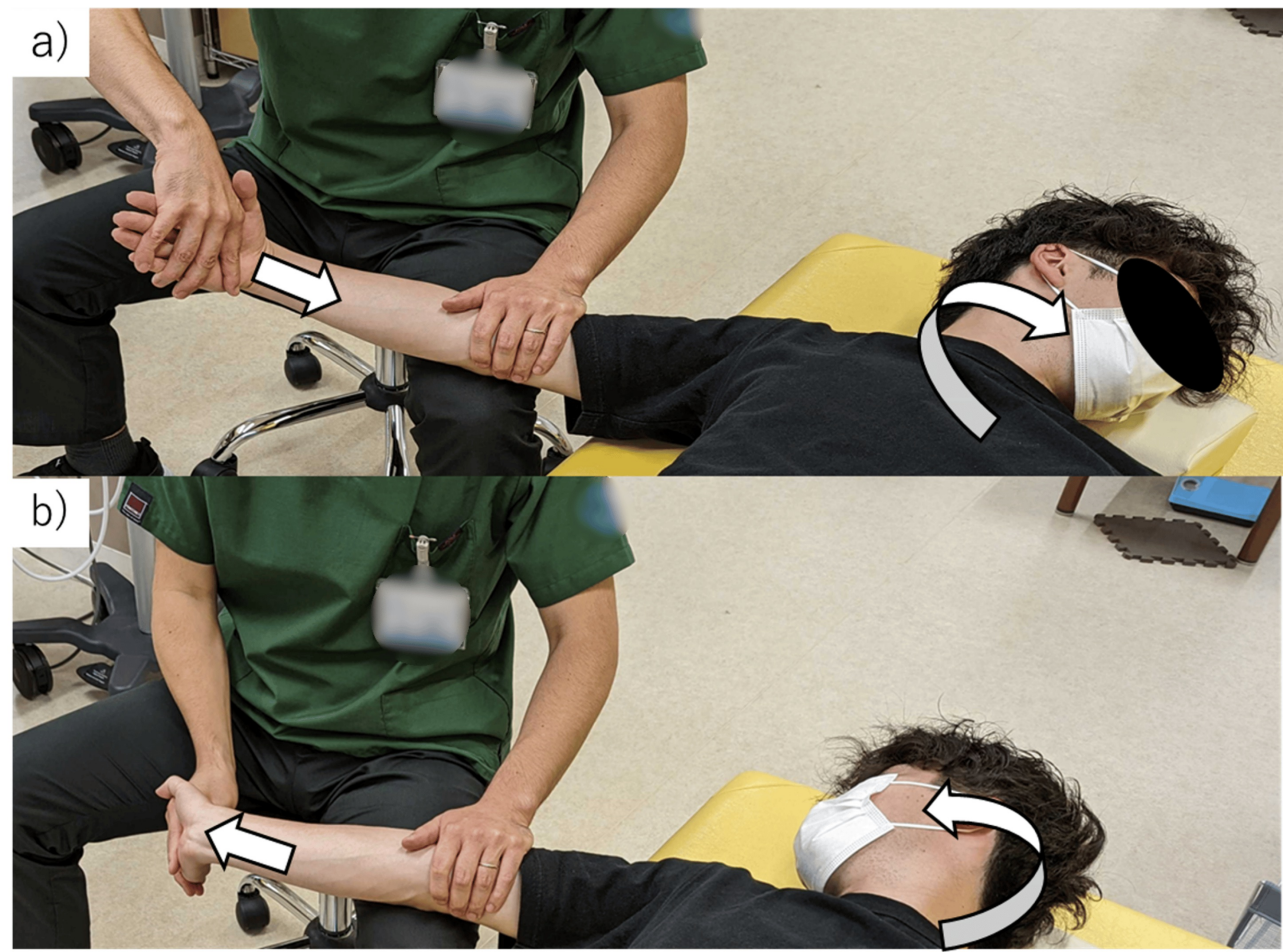
Median Nerve Gliding Exercises (a) Wrist extension combined with ipsilateral cervical side-bending; (b) wrist palmar flexion combined with contralateral cervical side-bending. Arrows indicate the direction of wrist and cervical movements during the exercises. These exercises were performed to facilitate smooth gliding of the median nerve and to reduce perineural tension, thereby contributing to symptom relief. This image was obtained from a healthy volunteer model for illustrative purposes. It does not represent the actual patient.

We acknowledge that standardized quantification of manual therapy parameters would further enhance reproducibility in future studies. To facilitate the dynamic excursion of the median nerve at the pronator teres level in the short-axis view, the operator introduced the fingers from the medial and lateral aspects of the pronator teres and applied a combination of targeted mobilization techniques, including precise short-axis manipulations and a controlled lift-off maneuver. All procedures were performed within pain tolerance to ensure procedural safety and comfort. In addition, median nerve gliding exercises were incorporated into the therapeutic regimen. The exercise sequence consisted of 90° shoulder abduction with elbow extension, followed by wrist extension combined with ipsilateral cervical side-bending, and then wrist palmar flexion with contralateral cervical side-bending. Each set comprised 10 to 15 controlled repetitions, performed within pain tolerance, in accordance with previously established protocols [12] (Figure 1, Video 5). Figure 1 illustrates the proper posture used during the nerve gliding exercises, emphasizing the coordinated movement between the wrist and cervical spine.

**Video 5:** Dynamic Behavior of the Median Nerve at the Pronator Teres Level The median nerve is visualized at the pronator teres level with the patient in a supinated forearm position. During wrist extension combined with ipsilateral cervical side-bending, the nerve glides distally, whereas during wrist flexion with contralateral cervical side-bending, it glides proximally. This dynamic ultrasound assessment demonstrates the physiologic excursion of the median nerve and highlights the utility of ultrasound in detecting potential motion restrictions at the entrapment site. For illustrative purposes, the demonstration was performed using a healthy male model, as no procedural video of the actual female patient was available.

The patient was also instructed to perform pronator teres self-massage and median nerve gliding exercises as part of a structured home-based exercise program designed to reinforce the therapeutic effects and promote sustained functional recovery.

Outcome

Symptomatic improvement was observed after the first session. After four treatment sessions over a two-month period, the NRS score decreased from 8/10 to 3/10. The patient was clinically monitored for approximately two months during the active treatment phase, after which no formal follow-up assessments were performed. Nevertheless, she continued her home-based exercise program and reported sustained symptom control and functional recovery informally during subsequent routine visits.

## Discussion

Ultrasound-guided hydrodissection in the present case likely improved median nerve excursion by mechanically releasing perineural restrictions. This is biomechanically plausible because cadaveric data demonstrate that hydrodissection reduces median nerve gliding resistance within the carpal tunnel, thereby facilitating nerve motion across constrained interfaces [13]. The immediate pain reduction observed after the first session in our patient is consistent with such a mechanism and may have amplified the effectiveness of the subsequent manual mobilization performed under real-time ultrasound visualization. Beyond immediate analgesia, ultrasound-guided hydrodissection may further contribute to symptom relief by reducing perineural adhesions and lowering median nerve gliding resistance, thereby facilitating physiologic neural excursion at the entrapment site [13]. Consistent with this mechanism, neural mobilization (gliding) has been associated with symptomatic and functional improvements in median neuropathies, although the certainty of evidence varies across studies [12]. In proximal median nerve entrapment, early case series of ultrasound-guided perineural hydrodissection have also demonstrated favorable outcomes, supporting the plausibility of this combined, mechanism-based approach in pronator teres syndrome [11].

Moreover, several patient-specific and diagnostic factors likely contributed to the favorable outcome. The patient’s occupation as a truck driver involved repetitive forearm use, including frequent cargo loading and unloading, which may have predisposed her to the development of PTS through chronic mechanical stress [2]. In addition, a comprehensive physical examination enabled accurate differentiation of PTS from conditions with overlapping symptoms such as CTS and cervical radiculopathy [5-8]. These factors facilitated precise identification of median nerve entrapment at the pronator teres level, which in turn guided a pathophysiology-based treatment strategy.

Beyond biomechanical plausibility and diagnostic accuracy, accumulating clinical evidence supports the therapeutic components used in this case. A systematic review in CTS reported symptomatic and functional improvements with neural mobilization, albeit with variable certainty across trials [12]. In our patient, combining ultrasound-guided hydrodissection and ultrasound-guided manual mobilization with a structured home-based exercise program (including nerve gliding) likely promoted sustained symptom control by reinforcing restored nerve mobility and reducing recurrent interface loading. Furthermore, early reports specifically in PTS have described favorable outcomes after ultrasound-guided perineural hydrodissection, supporting the applicability of image-guided perineural release to proximal median nerve entrapment [11]. Taken together, our results align with this literature and extend it by illustrating how real-time ultrasound can integrate interventional release, targeted manual therapy, and home-based exercise into a coherent, mechanism-driven care pathway for chronic, occupation-related PTS.

Contextualized against historical management, these findings are clinically meaningful. Classical series emphasized diagnostic heterogeneity and variable outcomes with surgical decompression for PTS [5-9]. By contrast, the present case suggests that an image-guided, minimally invasive approach coupled with rehabilitation may offer rapid symptom relief and functional recovery while potentially avoiding surgery, particularly when occupational loading and precise entrapment level are addressed upfront through careful examination and ultrasound guidance [1,2,11-13].

Given the descriptive and uncontrolled nature of this single-case report, the observed improvement should not be interpreted as definitive evidence of causality. Rather, it indicates a possible association between the multimodal intervention and symptom improvement, which warrants validation through larger, controlled studies.

This study has several limitations. First, as a single case report with a relatively short follow-up and no control group, the findings cannot be generalized to all patients with PTS; the durability of symptom relief remains uncertain, and causality cannot be definitively established. The relatively short follow-up period of two months limits conclusions about the long-term durability of the improvement. Second, as summarized in Table 1, several secondary clinical measures were not repeated at follow-up: provocative tests that were negative at baseline (such as Jackson, Spurling, Phalen, and carpal compression) and grip strength were omitted in routine care because the patient exhibited marked symptomatic improvement (NRS decreased from 8/10 to 3/10) and resolution of localized provocative findings at the pronator teres level. This pragmatic approach reflects routine practice, but systematic reassessment in future studies would more comprehensively document treatment effects. Third, validated patient-reported outcome measures (PROMs, such as the Disabilities of the Arm, Shoulder and Hand (DASH) or QuickDASH) were not obtained at baseline or follow-up; thus, functional changes were evaluated mainly using the NRS and clinical examination. Finally, several figures and a video were obtained from a healthy volunteer model because no recordings were available during the actual intervention, and no patient-identifiable data were retained or disclosed for ethical reasons. While these materials help clarify the procedure, they may not fully capture the patient’s condition.

Additionally, as blinding was not applicable in this single-patient case, assessment bias cannot be completely excluded despite the use of standardized procedures and consistent ultrasound evaluation. Further prospective studies with larger sample sizes are warranted to validate these preliminary findings and establish the long-term effectiveness and safety of ultrasound-guided hydrodissection combined with exercise-based rehabilitation in PTS.

## Conclusions

This case suggests that ultrasound-guided hydrodissection, when combined with ultrasound-guided manual mobilization and a structured home-based exercise program, may provide rapid symptom relief and facilitate functional improvement in pronator teres syndrome. These findings should be interpreted within the descriptive context of a single-case report.

The favorable outcome highlights the importance of considering occupational risk factors, performing accurate differential diagnosis from overlapping conditions such as CTS and cervical radiculopathy, and utilizing real-time ultrasound not only for diagnosis but also for guiding interventions. From a clinical perspective, this report suggests that a multimodal, minimally invasive approach may represent a practical and effective option before resorting to surgery in carefully selected patients. Although limited to a single case, this study underscores the need for future prospective research with larger cohorts, long-term follow-up, and direct comparisons with surgical or pharmacological interventions to establish the role of this strategy in routine clinical practice.
